# Clinical Characteristics, Patient-Reported Outcomes, and Previous Therapeutic Management of Patients with Uncontrolled Neuropathic Pain Referred to Pain Clinics

**DOI:** 10.1155/2014/518716

**Published:** 2014-05-05

**Authors:** José de Andrés, José-Luis de la Calle, María Pérez, Vanessa López

**Affiliations:** ^1^Anesthesia, Critical Care, and Multidisciplinary Pain Management Department, Valencia University General Hospital, Valencia University Medical School, Valencia, Spain; ^2^Pain Unit, Hospital Ramón y Cajal, Madrid, Spain; ^3^Medical Unit, Pfizer España, Avenida de Europa, 20 B Parque Empresarial La Moraleja, 28108 Alcobendas, Madrid, Spain

## Abstract

*Background.* The aim of this report was to evaluate the clinical profile and previous management of patients with uncontrolled neuropathic pain who were referred to pain clinics. *Methods.* We included adult patients with uncontrolled pain who had a score of ≥4 in the DN4 questionnaire. In addition to sociodemographic and clinical data, we evaluated pain levels using a visual analog scale as well as anxiety, depression, sleep, disability, and treatment satisfaction employing validated tools. *Results.* A total of 755 patients were included in the study. The patients were predominantly referred to pain clinics by traumatologists (34.3%) and primary care physicians (16.7%). The most common diagnoses were radiculopathy (43%) and pain of oncological origin (14.3%). The major cause for uncontrolled pain was suboptimal treatment (88%). Fifty-three percent of the patients were depressed, 43% had clinical anxiety, 50% rated their overall health as bad or very bad, and 45% noted that their disease was severely or extremely interfering with their daily activities. *Conclusions.* Our results showed that uncontrolled neuropathic pain is a common phenomenon among the specialties that address these clinical entities and, regardless of its etiology, uncontrolled pain is associated with a dramatic impact on patient well-being.

## 1. Introduction

Neuropathic pain is defined as pain that originates from a lesion or disease that affects the somatosensory pathways within the peripheral or central nervous system [1]. Neuropathic pain is common among the general population, with prevalence rates of 7-8% [2, 3]. Neuropathic pain, which is also a common occurrence (12%) among patients who are managed by primary care physicians [4], accounts for a high proportion (20%) of the patients who are referred to specialized pain units [5]. The causes of neuropathic pain comprise a wide and heterogeneous number of clinical conditions, such as diabetic neuropathy, complex regional pain syndrome, spinal cord injury pain, postherpetic neuralgia, postoperative pain, trigeminal neuralgia, drug-induced polyneuropathies, HIV-associated neuropathy, multiple sclerosis, and central pain syndromes secondary to vascular lesions [6, 7]. Although it may be acute in nature, in the vast majority of patients, neuropathic pain is a chronic condition. Regardless of its etiology, neuropathic pain is disabling and substantially impairs patients' health-related quality of life [8–12]. This condition is associated with high societal costs because of the patients' loss of productivity and increased utilization of health resources [8, 10, 13–15].

Managing neuropathic pain requires an interdisciplinary approach in which pharmacological treatment is fundamental [5, 16]. Despite the availability of several effective drugs, neuropathic pain treatment is challenging: response to treatment is unpredictable; despite the fact that the patients may receive several drugs for pain treatment, moderate-to-severe levels of pain are common; suboptimal treatment is also common with patients who receive ineffective treatments, such as nonsteroidal anti-inflammatory drugs or lower-than-recommended doses of the prescribed treatment; and delayed referral to pain clinics is also common [4, 8, 16–18].

Although there is consensus that many patients with neuropathic pain do not respond adequately or are unable to tolerate existing treatments [19, 20], epidemiologic information about this population is limited [21]. The aim of this report was to evaluate the clinical profile and previous management of patients with uncontrolled neuropathic pain who were referred to pain clinics.

## 2. Patients and Methods

### 2.1. Study Design, Setting, and Patients

This report was an observational, multicenter, and prospective study performed by 161 investigators from pain clinics throughout Spain between February 2009 and February 2010. The study was approved by the Ethics Committee of the Hospital General Universitario de Valencia (Spain). Written informed consent was obtained from every subject. The study was conducted in accordance with the principles of the Declaration of Helsinki. In this report, we presented the baseline (cross-sectional) data of the study.

For inclusion in the study, the patients had to fulfill the following criteria: age 18 years or older; referral to a pain clinic because of uncontrolled pain; and a score of equal to or greater than 4 in the DN4 questionnaire. The patients were excluded from the study if they were unable to understand the study objectives or complete the self-administered questionnaires.

### 2.2. Study Assessments

At baseline, the following information was recorded: sociodemographic data, type of specialist referring the patient, diagnostic confirmation of neuropathic pain, confirmation of the presence of uncontrolled pain as assessed by the investigator, etiology and duration of pain, causes for uncontrolled pain, pain intensity as measured using a 0 to 100 mm visual analog scale (VAS), and signs and symptoms of neuropathic pain. We also recorded pharmacological and nonpharmacological treatment for neuropathic pain. Additionally, the Spanish validated versions of the following questionnaires and scales were completed: DN4 questionnaire, Hospital and Anxiety Depression Scale (HADS), the Medical Outcomes Study Sleep (MOS Sleep) Scale, the World Health Organization Disability Assessment Schedule (WHO-DAS II), and the Treatment Satisfaction with Medicines Questionnaire (SATMED-Q).

The neuropathic pain diagnostic questionnaire DN4 consists of 10 items that describe different pain characteristics. A score of at least 4 of 10 possible points is considered acceptable to identify neuropathic pain with 83% sensitivity and 90% specificity [22–24].

The HADS, which is a self-administered instrument, consists of 14 items: 7 items that refer to depression symptoms and 7 items that refer to anxiety symptoms [25, 26]. Each item score ranges from 0 to 3, where 0 represents the absence of that symptom and 3 represents the highest severity or frequency of the symptom. By adding the 7 items of each subscale, two scores ranging from 0 to 21 are obtained that represent depression and anxiety (HADS-D and HADS-A), respectively.

The MOS Sleep Scale, a self-administered questionnaire, evaluates the key aspects of sleep [27, 28] and consists of 12 items that comprise six subscales or domains: sleep disturbances, snoring, shortness of breath or headache upon awakening, adequacy of sleep, day somnolence, and amount of sleep. Additionally, the MOS Sleep Scale provides a summary index of sleep disturbances that can be obtained from 9 of its items; the higher the score is, the worse the sleep is, with the exception of amount of sleep and adequacy of sleep dimensions, which are scored in the opposite direction. In patients with neuropathic pain, this scale has shown to have appropriate psychometric properties [28].

The WHO-DAS II comprises 12 items that evaluate an individual's level of functioning and disability in six areas: understanding and communicating, getting around, self-care, getting along with people, life activities, and participation in society [29–31]. The patients are required to answer questions regarding how many difficulties they experienced in the last 30 days as a result of their health condition, using a five-point scale from 1 (none) to 5 (extreme difficulty or cannot do it). The raw scores are transformed into a standard scale that ranges from 0 to 100; the higher scores reflect more severe disability. A global score is obtained that ranges from 0 to 700 (if work activities outside the home are assessed) or from 0 to 600.

The SATMED-Q, a self-administered questionnaire, consists of 17 items that evaluate six dimensions: treatment effectiveness, convenience of use, impact on daily activities, medical care, global satisfaction, and undesirable side-effects [32, 33]. The questionnaire also provides a global score for satisfaction with drug treatment by summing the scores of all of the domains. The raw scores are transformed into a scale that ranges from 0 to 100; the higher scores indicate greater satisfaction. Questions on medication side-effects include whether the patient experienced side-effects and whether the side-effects interfered with their physical exercises, leisure time, and daily activities.

### 2.3. Statistical Analysis

The analysis was essentially descriptive using the means and standard deviations for quantitative variables and using the absolute and relative frequencies for qualitative variables. The patients were categorized as having clinical anxiety or depression if they had a score equal or greater than 11 in the anxiety or depression subscales of the HAD.

## 3. Results

We included 755 patients in the study. We excluded 27 (3.6%) patients who did not meet the selection criteria, thereby leaving 728 evaluable patients.

### 3.1. General Characteristics of Patients

**Figure 1:**
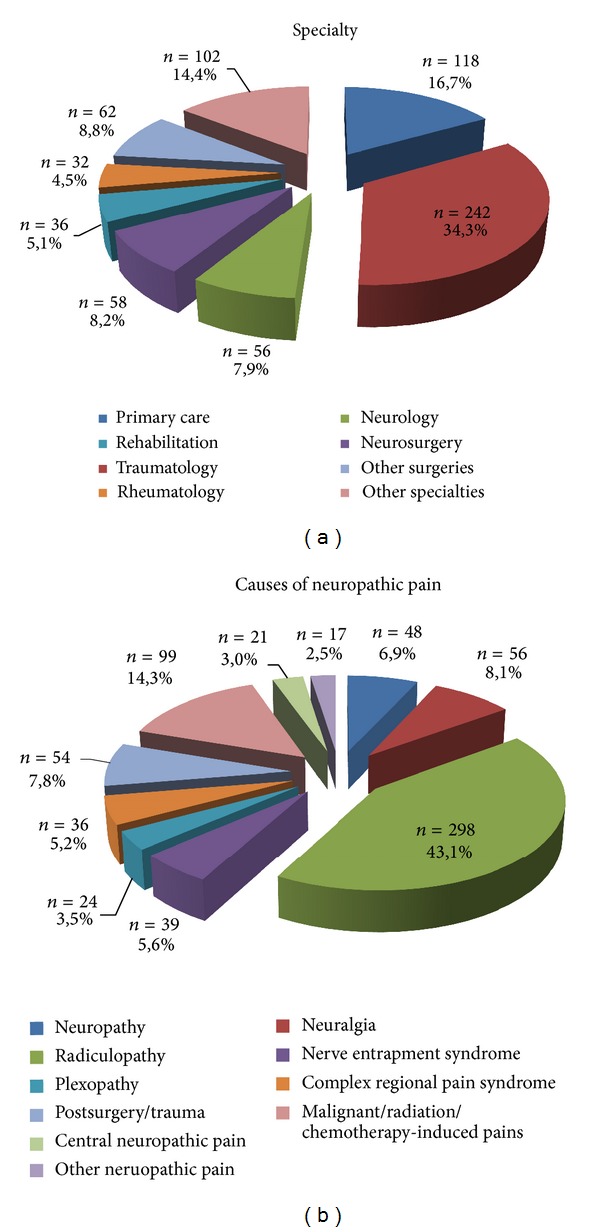
Specialists referring patients and clinical entities referred to pain clinics.

The patients had a mean age of 57 years, were predominantly women (61%), and exhibited obesity in a high proportion (20%) (Table 1). More than one-third of the patients were referred to pain clinics by traumatologists, followed by those who were referred by primary care physicians (Figure 1).

### 3.2. Pain Characteristics and Treatment

The patients had severe pain with a mean score of 75 using the VAS; the duration of pain was generally 2,6 years and was longer for those patients who were referred to pain clinics from the departments of rheumatology, neurosurgery, and neurology (Table 2). The majority (43%) of the patients were diagnosed with radiculopathy (Figure 1), which was the predominant diagnosis among those patients referred by traumatology, neurosurgery, rheumatology, and rehabilitation departments (Table 2). The patients who were referred by neurologists had trigeminal neuralgia and central neuropathic pain as their primary diagnoses, whereas primary care physicians referred patients with oncological pain or radiculopathy. The type, spontaneous or evoked, and subtypes of pain by clinical entity are presented in Table 3. All subtypes of spontaneous pain were present in more than 80% of the patients, regardless of the clinical entity. The subtypes of evoked pain were less represented, especially thermal allodynia; however, nearly every subtype was present in more than two-thirds of the patients in every clinical entity.

The major cause for uncontrolled pain, as assessed by the pain clinic investigators, was attributed to suboptimal treatment (88%) because of the use of ineffective drugs or subtherapeutic doses (Table 2). The use of ineffective drugs was less common among the patients who were referred by the neurology and neurosurgery departments.

The patients were receiving a mean of 3 drugs; one-third of the patients, regardless of the referral specialty, were receiving 4 or more drugs (Table 4). The most common prescribed drugs were antiepileptics (54%), opiods (40%), and nonsteroidal anti-inflammatory agents (40%); the latter drugs were more commonly prescribed by specialists in rehabilitation facilities (55%), rheumatologists (50%), and traumatologists (46%) and were less commonly prescribed by neurologists (28%) and primary care physicians (34%).  Table 5 shows the doses and treatment duration of the most common drugs that patients were receiving at the time of referral.

Patients with uncontrolled pain showed low satisfaction with their treatment with a global satisfaction score of 44 of 100. Treatment effectiveness and the impact of medicine on their everyday life were the areas exhibiting lower satisfaction, with mean scores of 32 and 30, respectively (Table 6).

The number of drugs that the patients were receiving was higher among the patients with central neuropathic pain (3.7), plexopathy (3.5), radiculopathy (3.3), and complex regional pain syndrome (3.2) and was lower among those with diabetic neuropathy (2.3) (Table 7). The use of antiepileptics, especially carbamazepine and oxcarbazepine, was higher in patients with trigeminal neuralgia (90.6%); the use of opioids was higher in patients with plexopathy (50%), nerve entrapment syndrome (46%), and radiculopathy (45%). Approximately 50% of the patients with radiculopathy, nerve entrapment syndrome, or complex regional syndrome were receiving NSAIDs. Suboptimal treatment was the major cause for uncontrolled pain, regardless of the clinical entity, but was overrepresented in patients with plexopathy (96%) and in patients with radiculopathy (91%) (Table 7). Overall, the treatment satisfaction was low, and the satisfaction with treatment efficacy was equally low (Table 7).

### 3.3. Impact of Uncontrolled Pain on Psychological Well-Being and Disability

More than half of the patients were diagnosed with depression, and 43% of the patients were diagnosed with anxiety (Table 8). Sleep was also deeply affected among these patients. The patients who were referred to pain clinics by rheumatologists exhibited symptoms that had the greatest impact on their psychological well-being and sleep (Table 8).

The proportion of disability among patients with uncontrolled pain was high, with approximately 50% of the patients rating their overall health as bad or very bad and 45% noting that their disease was severely or extremely interfering with their life (Table 9). The difficulties were present most of the time (22 of 30 days) and prevented them from executing their daily activities 15 days a month. The most affected dimensions were life activities either at home or at work (Table 9).

## 4. Discussion

Our results indicate that uncontrolled neuropathic pain is a problem that extends into multiple specialties that address the issue of chronic pain. Uncontrolled neuropathic pain appears to affect patients with clinical entities that are a subsidiary of traumatological care, in which radiculopathy is the most common cause. Other common causes of uncontrolled pain are pain of oncological origin and trigeminal neuralgia. Regardless of the cause and the specialist managing the patient with uncontrolled pain, we observed the following. The impact of the disease is high in terms of disrupting the patients' psychological well-being and disability; the major cause of uncontrolled pain is receiving suboptimal treatment; and the patients are generally dissatisfied with the treatment received.

Traumatologists were by far the primary referral specialists of uncontrolled pain (34%), and radiculopathy was the most common cause (43%) of uncontrolled pain. These findings are consistent with the most common causes of chronic pain in the general population. Thus, according to the survey of pain in Europe, the most frequent location of chronic pain was back pain (42%) and the more frequent cause of pain was herniated or deteriorated discs (15%) and traumatic injuries (12%) [34]. These results overlap with those reported in a previous study on the etiology of neuropathic pain in patients who attended pain clinics in Spain [13]. Although radiculopathy is the most common cause of uncontrolled pain because of its high prevalence as a cause of chronic pain, the presence of trigeminal neuralgia and pain of oncological origin as other common causes of uncontrolled pain is likely due to their refractoriness to current treatments. It is important to note that patients with uncontrolled oncological pain (i.e., pain of malignant origin, radiotherapy- or chemotherapy-induced) were primarily referred by surgical specialties and, surprisingly, by primary care physicians. The management of cancer pain requires the involvement of specialists of multiple disciplines, and anesthesiologists play a key role [35]. Therefore, we would have expected a higher rate of referrals of patients with oncological pain from specialties other than primary care.

The fact that neuropathic pain may be associated with the presence of anxiety and depressive symptoms and sleep disturbances is well known [36–40]. However, it should be emphasized that high proportions of depression (53%) and anxiety (43%) were found in our sample of patients with uncontrolled pain. Anxiety, depressive symptoms, and sleep disturbances are interrelated in patients with neuropathic pain and may increase the severity of pain [41–43] and contribute to the persistence of neuropathic pain [44]. Therefore, the management of uncontrolled pain should focus not only on the treatment of the underlying disease and its associated pain but also on the appropriate management of the accompanying anxiety, depressive symptoms, and sleep disturbances. Our results emphasize the enormous impact of uncontrolled pain on the patients' daily life. Half of the patients rated their overall health as bad or very bad, and 45% noted that their condition severely or extremely interfered with their life. The interference was high, regardless of the specialty or the pain etiology. However, regarding the etiology, the interference was higher for the complex regional pain syndrome and central neuropathic pain. We believe that this higher interference in these clinical entities more closely correlated with the underlying disease than to the pain itself because, with the exception of complex regional pain syndrome, the etiologies associated with the highest pain intensity are not those associated with the highest interference. The difficulties associated with uncontrolled pain were present most days, and the most severe impact was on daily activities at home or at work, that is, being totally unable to conduct their usual activities half of the days in the previous month. Although it was not specifically evaluated in our study, as it had been described for neuropathic pain in general [13, 14, 45], this degree of disability is likely to contribute to the high costs associated with neuropathic pain.

The investigators from the pain clinics determined that the main reason for uncontrolled pain was because the patients were receiving suboptimal treatment; that is, the patients received either ineffective drugs or subtherapeutic doses of the drugs. For instance, NSAIDs were used to treat more than 40% of the patients, and carbamazepine and oxcarbazepine, the first-line treatments for trigeminal neuralgia [16], were only used by 56% of the patients with that condition. However, regarding the latter drug, it is possible that those patients were receiving a second- or third-line treatment including surgery. Despite NSAIDs are considered ineffective and are not recommended for treating neuropathic pain [16, 19, 20, 46, 47], their use is common in patients with these conditions as it has been reported in several studies from different countries [18, 48, 49]. Noteworthy, in our study NSAIDs were used at high doses (e.g., over 1,400 mg/day of ibuprofen). The use of NSAIDs contributes to the suboptimal treatment of neuropathic and increases the risk of experiencing important adverse reactions. Suboptimal treatment has been previously described in patients with neuropathic pain who attended primary care clinics [4, 18, 50, 51]. According to our results, suboptimal treatment seems to affect all of the specialties involved in the care of patients with neuropathic pain. Treatment satisfaction was poor across several specialties and clinical entities. The areas that were rated with lower satisfaction were treatment effectiveness and impact on daily activities, and these areas are closely related. Although satisfaction with medical care received a higher rating than impact on daily activities, the former issue seemed to be an area for improvement. Despite the fact that most patients experienced treatment side-effects according to the SATMED-Q, medical treatment was rated with greater satisfaction; however, it should be noticed that acute side effects were not reflected in that evaluation.

The major limitation in our study was the definition of uncontrolled pain that was entirely based on the subjective evaluation of the investigators from the pain clinics. We accepted this subjective evaluation because there is no standard definition of refractory neuropathic pain [21]. Recently, a group of experts tried to achieve consensus on this matter [52]; according to that consensus, to classify a neuropathic pain as refractory, “it should have had a trial of treatment with at least four drugs of known effectiveness, each drug should have been tried at least three months or until side effects prevent adequate dosage, and despite the above treatment, the intensity of pain should have reduced by less than 30%, should remain at a level of at least 5 on a 0–10 scale, and/or should continue to contribute significantly to poor quality of life” [52]. Because the patients in our study were considered to have received suboptimal treatment, it is difficult to meet those criteria. However, the patients' pain was clearly persistent, severe, disabling, and, therefore, uncontrolled.

Overall, our results showed that uncontrolled neuropathic pain is a common phenomenon among the specialties that address these clinical entities, and regardless of its etiology, uncontrolled neuropathic pain is associated with a dramatic impact on patient well-being. A definite need exists for improving the management of neuropathic pain in all of these specialties, which should not be limited to the improvement of pain but also should be extended to the management of psychological symptoms and possibly to the improvement of the doctor-patient relationship.

## Figures and Tables

**Table 1:** Sociodemographic characteristics for the overall simple and referring specialty.

Characteristic	Overall *N* = 728	Primary care *N* = 118	Traumatology *N* = 242	Neurology *N* = 56	Neurosurgery *N* = 58	Rehabilitation *N* = 36	Rheumatology *N* = 32	Other surgeries *N* = 62	Other specialties *N* = 102
Age, mean ± SD	57.2 ± 15.2	63.3 ± 13.6	56.2 ± 14.2	54.9 ± 14.9	54.3 ± 12.0	53.0 ± 14.8	55.5 ±13.1	57.3 ± 13.7	61.5 ± 13.5
Sex (female), %	60.6	56.3	65.8	60.7	63.0	51.4	78.1	59.7	51.0
BMI, %									
Underweight	1.6	0.0	1.5	8.5	0.0	3.0	6.9	0.0	0.0
Normal	34.4	31.8	31.2	36.2	36.4	39.4	27.6	43.1	40.2
Overweight Obesity I	43.9	51.4	43.7	38.3	47.7	36.4	41.4	37.3	45.1
Obesity II	16.7	15.0	19.6	12.8	11.4	18.2	17.2	17.6	11.0
Obesity III	2.1	0.9	2.5	4.3	2.3	0.0	3.4	2.0	2.4
Obesity IV	1.3	0.9	1.5	0.0	2.3	3.0	3.4	0.0	1.2

BMI: body mass index; SD: standard deviation.

**Table 2:** Pain characteristics.

Characteristic	Overall *N* = 728	Primary care *N* = 118	Traumatology *N* = 242	Neurology *N* = 56	Neurosurgery *N* = 58	Rehabilitation *N* = 36	Rheumatology *N* = 32	Other surgeries *N* = 62	Other specialties *N* = 102
DN4 (0–10), mean ± SD	6.6 ± 1.6	6.6 ± 1.6	6.5 ± 1.5	7.1 ± 1.8	6.7 ± 1.5	7.3 ± 1.7	6.3 ± 1.5	6.5 ± 1.5	6.7 ± 1.6
VAS, mean ± SD	74.5 ± 15.3	73.7 ± 15.5	76.6 ± 13.5	75.8 ± 15.8	73.9 ± 15.1	76.0 ± 12.2	70.9 ± 12.6	72.3 ± 18.6	73.0 ± 17.3
Duration of pain (yrs), mean ± SD	2.6 ± 3.6	2.2 ± 3.1	2.5 ± 3.5	3.7 ± 4.4	4.3 ± 5.3	3.3 ± 3.0	4.4 ± 5.6	1.2 ± 1.5	1.7 ± 2.3
*Causes for uncontrolled pain, % *									
Misdiagnosis	11.1	11.9	12.8	3.6	5.2	12.7	9.4	16.1	8.3
Nonoptimized treatment	87.6	82.2	93.4	82.1	87.9	83.3	90.6	88.7	83.3
Use of ineffective drugs	45.8	45.9	50.4	28.3	37.3	46.7	41.4	52.7	44.3
Subtherapeutic dose	45.7	48.5	46.0	47.8	43.1	56.5	41.4	30.9	50.0
Other	25.3	18.6	23.5	37.0	39.2	20.0	24.7	21.8	31.0
Lack of compliance	7.2	5.1	5.9	12.5	5.2	11.1	15.6	4.8	6.2
Intolerance	6.6	9.3	3.3	16.1	6.9	2.8	9.4	3.2	7.8
Other	7.3	8.5	9.8	10.7	6.9	11.1	3.1	8.1	4.1
*Causes of neuropathic pain, % *									
Neuropathy									
Diabetic	3.8	10.4	0.9	5.8	0.0	2.8	0.0	3.5	6.1
Other	3.2	0.9	0.9	5.8	1.8	2.8	9.4	8.8	0.0
Neuralgia									
Trigeminal	4.6	7.0	0.4	25.0	5.5	0.0	4.2	5.3	0.0
Other	3.5	2.6	2.1	7.7	3.6	2.8	3.3	3.1	8.8
Radiculopathy	43.1	32.5	67.0	11.5	63.6	52.8	63.3	8.3	3.5
Nerve entrapment syndrome	5.6	4.4	2.1	0.0	5.5	2.8	16.7	1.8	9.4
Plexopathy	3.5	3.5	3.9	3.8	0.0	5.2	3.3	1.8	2.8
Complex regional pain syndrome	5.2	0.9	8.6	5.8	3.6	16.7	3.3	1.8	1.0
Postsurgery/trauma	7.8	3.5	3.0	1.9	5.5	2.8	3.3	11.5	45.6
Malignant/radiation/chemotherapy-induced pain	14.3	36.0	1.3	11.5	5.5	0.0	3.3	41.7	8.8
Central neuropathic pain	3.0	0.9	1.3	17.3	5.5	11.1	0.0	1.0	0.0
Other neuropathic pains	2.5	1.8	1.3	3.8	0.0	2.1	3.3	10.5	2.8

DN4: neuropathic pain diagnostic questionnaire; SD: standard deviation; VAS: visual analog scale; Yrs: years.

**Table 3:** Type of pain by clinical entity.

Characteristic	Diabetic neuropathy *N* = 26	Other neuropathies *N* = 22	Trigeminal neuralgia *N* = 32	Other neuralgia *N* = 24	Radiculopathy *N* = 298	Nerve entrapment syndrome *N* = 39	Plexopathy *N* = 24	Complex regional pain syndrome *N* = 36	Postsurgery/trauma *N* = 54	Oncological pain *N* = 99	Central neuropathic pain *N* = 21
Spontaneous pain, %											
Lancinating	98.0	89.5	96.6	100.0	60.1	91.2	81.0	87.5	90.9	90.4	90.0
Burning	100.0	90.0	95.7	92.6	87.0	97.1	82.7	96.9	89.9	92.5	90.0
Paraesthesias	100.0	95.0	86.2	82.6	96.8	97.1	90.9	93.7	93.3	90.2	90.5
Dysaesthesias	100.0	95.0	89.3	91.3	92.8	94.1	95.5	100.0	95.5	94.4	85.0
Evoked pain, %											
Static allodynia	95.8	90.0	91.5	88.3	68.7	73.5	54.5	93.6	84.8	82.0	80.0
Dynamic allodynia	91.7	95.0	86.2	78.3	69.6	70.6	54.5	100.0	93.5	83.7	85.0
Thermal allodynia	70.8	60.0	85.2	68.2	52.0	61.8	45.5	78.1	65.9	71.5	75.0
Mechanical hyperalgesia	84.0	85.0	89.3	78.3	81.2	73.5	77.3	90.6	86.7	86.8	71.4
Hyperpathia	83.3	70.0	77.8	69.6	70.3	63.6	72.7	84.4	76.7	77.5	73.7

**Table 4:** Treatment of neuropathic pain by specialty.

Characteristic	Overall *N* = 728	Primary care *N* = 118	Traumatology *N* = 242	Neurology *N* = 56	Neurosurgery *N* = 58	Rehabilitation *N* = 36	Rheumatology *N* = 32	Other surgeries *N* = 62	Other specialties *N* = 102
*Number of previous treatments *									
Mean ± SD	3.13 ± 1.51	2.99 ± 1.44	3.21 ± 1.57	3.39 ± 1.56	3.10 ± 1.18	3.09 ± 1.28	3.10 ± 1.37	2.76 ± 1.49	3.13 ± 1.72
1, %	11.9	15.5	12.7	5.6	5.8	9.1	3.3	17.6	10.9
2, %	27.4	23.6	24.9	27.8	28.8	27.3	43.3	35.3	31.5
3, %	25.6	29.1	24.9	27.8	30.8	27.3	20.0	21.6	27.2
>4, %	35.1	31.8	37.6	38.9	34.6	36.4	33.3	25.5	30.4
*Treatment at the time of inclusion, % *									
Paracetamol	25.0	19.1	33.9	13.0	15.4	18.2	26.7	25.5	22.8
NSAIDs	40.1	33.6	47.5	27.8	36.5	54.5	50.0	43.1	25.0
Opiods	39.6	41.8	40.3	38.9	55.8	33.3	53.3	17.6	35.9
Tramadol	29.5	28.2	29.4	29.6	48.1	24.2	43.3	13.7	28.3
Others	14.8	16.4	14.9	18.5	15.4	9.1	6.7	7.8	15.2
AED	54.1	56.4	48.4	81.5	69.2	48.5	43.3	41.2	56.5
Pragabalin	26.7	21.8	29.4	33.3	28.8	24.2	23.3	23.5	25.0
Gabapentin	23.5	28.2	19.0	31.5	34.6	15.2	10.0	17.6	30.4
Carbamazepine/oxcarbazepine	6.3	9.1	0.5	31.5	3.8	6.1	3.3	3.9	5.4
Other	4.4	2.7	3.2	7.4	11.5	6.1	6.7	0.0	5.4
Antidepressants	19.7	24.5	15.8	31.5	17.3	9.1	20.0	17.6	25.0
Duloxetine	5.3	5.5	5.4	1.9	5.8	3.0	10.0	3.9	6.5
Tricyclics	11.3	13.6	6.3	25.9	11.5	6.1	6.7	11.8	17.4
Other	4.2	5.5	4.1	5.6	1.9	3.0	10.0	2.0	3.3
Other drugs	47.6	47.3	46.6	51.9	34.6	39.4	46.7	51.0	54.3
Nonpharmacological	52.0	46.4	58.8	44.4	51.9	81.8	53.3	47.1	39.1

AED: antiepileptic drugs: NSAIDs: nonsteroidal anti-inflammatory drugs; SD: standard deviation.

**Table 5:** Most common (≥5%) drugs for the treatment of neuropathic pain at the time of referral.

Drug	% patients	Mean dose (SD) mg/day	Duration (months) mean (SD)
Paracetamol	21.2	2,398.6 (960.1)	5.5 (6.3)
NSAIDs			
Ibuprofen	17.9	1,428.4 (457.2)	8.5 (13.3)
Metamizol	14.8	2,158.8 (1,598.7)	8.5 (13.3)
Diclofenac	6.3	124.9 (34.8)	8.5 (13.3)
Opiods			
Tramadol	27.1	182.9 (110.1)	12.1 (21.5)
Fentanil	8.6	18.5 (25.1)	7.5 (12.9)
AED			
Pragabalin	27.2	244.6 (242.5)	13.8 (17.4)
Gabapentin	19.0	1,297.7 (646.9)	9.3 (12.0)
Carbamazepine	5.1	577.1 (313.5)	22.2 (27.5)
Antidepressants			
Amitriptyline	10.8	36.2 (27.3)	12.9 (15.4)
Duloxetine	6.1	58.3 (22.0)	8.1 (8.0)
Other drugs			
Clonazepam	5.7	2.1 (2.7)	2.2 (1.0)

SD: standard deviation.

**Table 6:** Treatment satisfaction by specialty.

Characteristic	Overall *N* = 728	Primary care *N* = 118	Traumatology *N* = 242	Neurology *N* = 56	Neurosurgery *N* = 58	Rehabilitation *N* = 36	Rheumatology *N* = 32	Other surgeries *N* = 62	Other specialties *N* = 102
Experiencing side effects, %	51.7	57.8	49.3	67.9	51.9	47.1	60.0	37.3	46.3
SATMED-Q dimensions, mean ± SD									
Undesirable side effects	74.8 ± 29.2	72.34 ± 29.08	75.14 ± 28.24	70.12 ± 28.29	70.83 ± 33.14	73.51 ± 31.59	70.83 ± 31.99	84.76 ± 25.44	77.95 ± 30.55
Treatment effectiveness	32.4 ± 25.8	34.20 ± 25.72	31.93 ± 24.76	38.56 ± 28.58	32.27 ± 25.52	28.23 ± 25.52	39.03 ± 24.67	39.44 ± 30.54	24.24 ± 24.06
Convenience of use	58.2 ± 25.2	60.48 ± 25.32	59.91 ± 23.83	59.80 ± 24.82	53.01 ± 28.79	58.47 ± 26.23	62.22 ± 22.61	60.05 ± 24.73	52.06 ± 27.09
Impact on daily living/activities	30.9 ± 26.6	33.41 ± 26.05	29.46 ± 26.45	35.26 ± 27.45	33.83 ± 28.29	26.82 ± 24.39	34.72 ± 21.00	37.77 ± 30.19	24.64 ± 25.65
Medical care	63.9 ± 28.0	63.29 ± 29.55	62.50 ± 28.26	64.66 ± 26.86	65.25 ± 27.12	59.38 ± 26.94	55.83 ± 29.68	68.62 ± 29.66	68.41 ± 25.97
Global satisfaction	44.3 ± 30.3	50.23 ± 29.60	42.69 ± 30.49	50.16 ± 29.68	44.17 ± 29.08	33.59 ± 30.64	45.11 ± 28.04	52.13 ± 33.31	38.68 ± 28.75
Total composite score	50.7 ± 17.5	51.88 ± 17.18	50.92 ± 17.11	53.36 ± 18.09	48.88 ± 18.97	47.35 ± 18.98	52.32 ± 16.62	57.57 ± 20.96	46.37 ± 14.96

SATMED-Q: Treatment Satisfaction with Medicines Questionnaire; SD: standard deviation.

**Table 7:** Pharmacological treatment, reasons for uncontrolled pain, and treatment satisfaction by clinical entity.

Characteristic	Diabetic neuropathy *N* = 26	Other neuropathies N = 22	Trigeminal neuralgia N = 32	Other neuralgia N = 24	Radiculopathy *N* = 298	Nerve entrapment syndrome N = 39	Plexopathy N = 24	Complex regional pain syndrome N = 36	Postsurgery/trauma N = 54	Oncological pain N = 99	Central neuropathic pain N = 21
VAS, mean ± SD	77.5 ± 13.4	68.6 ± 15.6	79.8 ± 18.9	73.3 ± 20.0	74.3 ± 13.5	75.4 ± 13.2	77.7 ± 19.4	78.3 ± 9.7	70.7 ± 17.8	74.2 ± 17.7	72.7 ± 13.3
WHO-DAS II Interference with life (severe/extreme), %	52.0	52.7	42.9	39.1	46.7	38.3	52.4	67.7	33.4	30.4	66.7
Number of previous treatments, mean ± SD	2.30 ± 1.26	3.00 ± 1.50	2.87 ± 1.58	2.90 ± 1.48	3.32 ± 1.48	2.63 ± 1.48	3.45 ± 1.47	3.23 ± 1.57	2.85 ± 1.68	2.76 ± 1.23	3.65 ± 1.81
*Treatment at the time of inclusion, % *											
Paracetamol	17.4	5.6	6.3	23.8	32.0	28.6	22.7	20.0	25.5	20.9	10.0
NSAIDs	17.4	33.3	21.9	47.6	49.6	48.6	40.9	48.6	44.7	22.0	10.0
Opiods	34.8	27.8	28.1	23.8	44.9	45.7	50.0	37.1	27.7	31.9	50.0
Tramadol	21.7	22.2	28.1	14.3	33.5	34.3	45.5	25.7	14.9	24.2	35.0
Others	13.0	11.1	3.1	9.5	15.1	14.3	18.2	20.0	12.8	13.2	30.0
AED	52.2	72.2	90.6	47.6	45.6	42.9	45.5	54.3	36.2	70.3	85.0
Pragabalin	26.1	38.9	25.0	14.3	28.3	14.3	27.3	31.4	19.1	24.2	35.0
Gabapentin	30.4	33.3	31.3	19.0	16.9	25.7	18.2	11.4	17.0	38.5	60.0
Carbamazepine/ oxcarbazepine	0.0	0.0	56.3	14.3	1.1	0.0	4.5	5.7	0.0	12.1	10.0
Other	4.3	5.6	12.5	0.0	2.6	2.9	0.0	5.7	0.0	6.6	20.0
Antidepressants	17.4	22.2	18.8	19.0	16.2	14.3	45.5	28.6	14.9	23.1	20.0
Duloxetine	8.7	5.6	0.0	4.8	6.6	2.9	0.0	5.7	4.3	5.5	5.0
Tricyclics	8.7	22.2	15.6	9.5	5.9	8.6	31.8	20.0	8.5	16.5	5.0
Other	0.0	0.0	3.1	4.8	4.4	5.7	13.6	5.7	2.1	1.1	10.0
Other drugs	34.8	44.4	43.8	52.4	50.0	37.1	40.9	37.1	44.7	48.4	55.0
*Causes for uncontrolled pain, % *											
Misdiagnosis	15.4	18.2	3.1	12.5	8.4	10.3	20.8	11.1	27.8	9.1	4.8
Nonoptimized treatment	80.8	77.3	78.1	83.3	91.2	89.7	95.8	86.1	77.8	87.9	85.7
Use of ineffective drugs	61.9	29.4	20.0	45.0	48.7	57.1	39.1	48.4	69.0	36.8	11.1
Subtherapeutic dose	47.6	41.2	52.0	25.0	46.1	40.0	43.5	38.7	28.6	57.5	55.6
Other	9.5	41.2	44.0	40.0	24.4	17.1	30.4	25.8	19.0	21.8	50.0
Lack of compliance	15.4	4.5	0.0	12.5	6.1	7.7	4.2	11.1	1.9	5.1	9.5
Intolerance	0.0	9.1	12.5	4.2	7.7	5.1	4.2	2.8	3.7	6.1	19.0
Other	11.5	9.1	12.5	4.2	6.4	0.0	0.0	5.6	7.4	10.1	9.5
*SATMED-Q *											
Total score, mean ± SD	51.0 ± 16.1	52.2 ± 18.3	50.7 ± 15.6	56.5 ± 22.1	51.2 ± 17.2	53.8 ± 16.3	49.7 ± 18.1	42.1 ± 14.2	49.0 ± 21.3	48.2 ± 15.6	49.7 ± 19.1
Efficacy, mean ± SD	26.0 ± 23.9	28.2 ± 31.6	35.7 ± 25.7	33.7 ± 30.5	32.7 ± 24.7	37.6 ± 24.2	34.5 ± 30.3	23.9 ± 26.7	29.6 ± 27.3	30.5 ± 24.0	31.6 ± 26.1

AED: antiepileptic drugs; NSAIDs: nonsteroidal anti-inflammatory drugs; SATMED-Q: Treatment Satisfaction with Medicines Questionnaire; SD: standard deviation; VAS: visual analog scale; WHO-DAS II: World Health Organization Disability Assessment Schedule II; Yrs: years.

**Table 8:** Depression, anxiety, and sleep.

Characteristic	Overall *N* = 728	Primary care N = 118	Traumatology N = 242	Neurology N = 56	Neurosurgery N = 58	Rehabilitation N = 36	Rheumatology N = 32	Other surgeries N = 62	Other specialties N = 102
HADS-depression (0–21)									
mean ± SD	10.7 ± 4.6	10.2 ± 4.5	10.8 ± 4.2	10.5 ± 4.7	10.8 ± 4.8	10.5 ± 4.6	12.5 ± 4.3	9.2 ± 5.6	11.3 ± 5.1
Clinical depression, %	53.0	48.1	55.0	53.8	53.8	52.9	71.4	41.5	54.8
HADS-anxiety (0–21)									
mean ± SD	9.7 ± 4.2	9.0 ± 4.0	9.8 ± 3.8	9.9 ± 4.3	9.4 ± 4.4	9.8 ± 4.5	11.0 ± 3.8	9.9 ± 5.0	9.4 ± 4.6
Clinical anxiety, %	43.1%	32.4	45.4	44.2	34.6	51.4	63.3	43.4	43.5
MOS-Sleep, mean ± SD									
Summary index (0–100)	47.8 ± 19.1	43.5 ± 19.2	48.8 ± 18.5	50.0 ± 20.9	49.0 ± 21.1	45.4 ± 17.7	56.0 ± 12.1	45.4 ± 18.9	46.1 ± 20.0
Sleep disturbance (0–100)	51.8 ± 23.1	47.7 ± 23.1	53.8 ± 22.3	51.6 ± 24.7	52.1 ± 26.1	48.2 ± 24.7	63.1 ± 15.8	49.1 ± 22.5	49.7 ± 23.4
Snoring (0–100)	38.1 ± 30.4	33.3 ± 28.0	37.0 ± 30.5	37.1 ± 28.9	32.9 ± 28.8	44.1 ± 28.2	39.3 ± 33.2	40.4 ± 29.9	43.3 ± 34.7
Shortness of breath (0–100)	24.4 ± 25.8	22.7 ± 26.1	24.9 ± 24.8	28.9 ± 29.8	22.7 ± 27.2	25.1 ± 23.4	27.6 ± 27.0	20.0 ± 23.1	23.6 ± 27.5
Adequacy of sleep (100–0)	37.1 ± 27.5	43.7 ± 28.7	36.2 ± 26.5	35.8 ± 28.5	34.4 ± 29.0	39.1 ± 24.4	28.6 ± 21.8	36.0 ± 28.3	38.6 ± 29.1
Amount of sleep, hours	5.6 ± 1.7	6.0 ± 2.5	5.4 ± 1.4	5.7 ± 1.68	5.8 ± 1.5	5.7 ± 1.5	5.1 ± 1.0	5.5 ± 1.70	5.6 ± 1.7
Daytime somnolence (0–100)	39.5 ± 19.8	38.6 ± 18.0	38.7 ± 19.9	47.2 ± 21.7	38.7 ± 20.5	37.6 ± 20.7	43.1 ± 17.9	34.1 ± 18.2	40.7 ± 21.1

HADS: Hospital and Anxiety Depression Scale; MOS-Sleep: the Medical Outcomes Study Sleep Scale; SD: standard deviation.

**Table 9:** Disability (WHO-DAS II) by specialty.

Characteristic	Overall *N* = 728	Primary care N = 118	Traumatology N = 242	Neurology N = 56	Neurosurgery N = 58	Rehabilitation N = 36	Rheumatology N = 32	Other surgeries N = 62	Other specialties N = 102
WHO-DAS II H1-H5									
Overall health (Bad/very bad), %	48.9	44.9	49.6	52.7	56.6	45.7	53.5	36.4	51.0
Interference with life (Severe/extreme), %	45.3	36.3	49.3	51.0	47.0	44.1	50.0	31.5	46.8
Duration of difficulties (days), mean ± SD	22.4 ± 9.9	20.29 ± 11.07	23.00 ± 9.21	21.11 ± 9.97	22.10 ± 10.02	25.21 ± 8.06	22.07 ± 7.62	19.89 ± 11.95	23.55 ± 9.95
Days totally unable to carry out usual activities/work, mean ± SD	14.7 ± 11.7	11.71 ± 11.54	16.23 ± 11.18	13.83 ± 11.51	15.04 ± 11.29	16.35 ± 12.54	14.90 ± 11.54	13.47 ± 12.54	14.44 ± 12.50
Days cutting back or reducing usual activities/work	19.2 ± 11.1	15.76 ± 12.04	20.65 ± 10.29	17.00 ± 11.46	20.02 ± 10.51	20.47 ± 11.34	19.37 ± 10.33	17.72 ± 11.75	20.13 ± 11.33
WHO-DAS II dimensions, mean ± SD									
Understanding and communicating	40.0 ± 24.4	37.16 ± 23.79	40.12 ± 23.69	44.10 ± 26.24	40.80 ± 25.38	39.64 ± 22.17	50.83 ± 19.40	35.45 ± 26.11	38.82 ± 25.94
Getting around	57.5 ± 30.4	47.18 ± 31.97	64.96 ± 26.75	51.89 ± 32.74	62.74 ± 29.26	67.50 ± 31.24	66.25 ± 22.06	48.41 ± 29.07	48.05 ± 32.41
Self-care	38.6 ± 26.9	30.33 ± 24.23	42.49 ± 25.34	38.99 ± 31.17	38.99 ± 22.63	45.71 ± 23.69	39.44 ± 28.19	35.45 ± 29.58	35.79 ± 30.46
Getting along with people	33.5 ± 28.4	30.68 ± 28.87	34.64 ± 27.10	35.85 ± 30.43	34.91 ± 28.73	28.57 ± 25.10	43.33 ± 25.37	28.18 ± 27.24	33.97 ± 31.79
Life activities, household	67.2 ± 30.5	58.11 ± 34.73	72.54 ± 26.25	66.04 ± 33.65	70.75 ± 26.74	67.14 ± 26.96	75.00 ± 25.43	57.27 ± 31.06	63.68 ± 33.77
Life activities, work	66.2 ± 33.1	55.86 ± 36.14	72.87 ± 31.00	63.21 ± 32.74	74.53 ± 27.07	61.43 ± 34.48	63.79 ± 26.38	60.19 ± 35.53	63.16 ± 35.14
Participation in society	60.9 ± 23.1	56.31 ± 24.21	62.39 ± 22.15	63.52 ± 22.89	62.58 ± 23.55	57.62 ± 19.11	68.89 ± 17.36	52.42 ± 28.04	62.33 ± 22.72

SD: standard deviation; WHO-DAS II: World Health Organization Disability Assessment Schedule II.
